# Awareness, Understanding and HIV Stigma in Response to Undetectable = Untransmittable Messages: Findings from a Nationally Representative Sample in the United Kingdom

**DOI:** 10.1007/s10461-022-03710-9

**Published:** 2022-06-10

**Authors:** Rory Coyne, Jane C. Walsh, Chris Noone

**Affiliations:** School of Psychology, National University of Ireland, Galway, Ireland

**Keywords:** Human immunodeficiency virus, Undetectable = Untransmittable, U = U, Prospect theory, HIV stigma

## Abstract

‘Undetectable = Untransmittable’, or ‘U = U’, is a message which communicates the scientific consensus that people living with HIV who maintain an undetectable viral load cannot sexually transmit HIV to others. This research aimed to empirically test whether a protection-framed U = U message is more effective at decreasing HIV stigma and increasing perceived accuracy of U = U than a risk-framed message. A nationally representative UK sample (*N* = 707) completed an online experiment. Participants viewed one of two U = U messages (protection-framed or risk-framed) and completed an online questionnaire. No evidence of a difference in HIV stigma at post-test or in perceived accuracy of U = U was found between the two message frame conditions. A minority of participants were aware of U = U prior to participation. Post-intervention, the majority of participants rated U = U as at least somewhat accurate. Higher understanding of U = U was associated with lower post-test stigma following a protection-framed message. Following a brief intervention, among a sample predominantly unaware of U = U previously, there was an overall favourable rating of U = U. No evidence was found for an effect of message framing on HIV stigma or perceived accuracy of U = U, but participants who completed a pre-test measure of stigma rated U = U as less accurate.

## Introduction

The advent of highly active antiretroviral therapy (HAART) for Human Immunodeficiency virus (HIV) has resulted in numerous scientific breakthroughs and quality of life improvements for people living with HIV (PLHIV). HAART can suppress HIV to a level at which it can no longer be detected by regular laboratory tests, and consequently, in addition to allowing PLHIV to live long and healthy lives, their HIV cannot be sexually transmitted to others [1]. The slogan ‘Undetectable = Untransmittable’, or ‘U = U’ was debuted by the Prevention Access Campaign in the United States in 2016 to maximise dissemination of the established scientific consensus that undetectable HIV cannot be sexually transmitted to others. This consensus is supported by clinical trials which have demonstrated no linked HIV transmissions among mixed status couples, including the HIV Prevention Trials Network 052 [2], PARTNER Studies [3], and Opposites Attract [4]. It is hoped that awareness of U = U will challenge HIV-related stigma by framing undetectability as a positive sexual health indicator [5].

The U = U message is perceived as at least somewhat accurate by between 53.2% [6] and 80% [7] in surveys of gay, bisexual and other men who have sex with men (gbMSM). However, gbMSM are more likely to be aware of U = U messaging [8]. Far less is known regarding how U = U is received and perceived in the general population.

The stigma associated with HIV acts as a mechanism for driving the spread of HIV, through impeding engagement with treatment and testing services, as well as hindering knowledge provision [9]. In the wider context, HIV stigma also manifests on three levels: interpersonal (discriminatory behaviors towards people living with HIV), institutional (such as the enforcement of discriminatory laws surrounding HIV status disclosure), and internalised (such as feeling ashamed of one’s status) [10]. Rivera and colleagues [11] examined the association between U = U awareness and anticipated HIV stigma among HIV-negative heterosexually active men in the US. It was found that awareness of U = U was negatively associated with two forms of interpersonal stigma (dating-related and sex-related). These findings could indicate that tailored U = U messaging might reduce HIV stigma in its varying forms in the general population. This would be in line with evidence that information on both the health and prevention benefits of HAART has been shown to decrease stigmatising attitudes towards PLHIV [12].

However, whether the U = U message reduces HIV stigma in the general population has yet to be formally examined, and a recent systematic review called for exploration into the complementary factors which could improve the efficacy of U = U messaging [13]. Therefore, one knowledge gap within the literature concerns the underpinnings of U = U communication. Rendina and colleagues [6] recommended a shift in U = U message framing from language that focuses on risk reduction, to language that emphasises protection from HIV transmission. This shift in framing could enhance acceptability of U = U by making the protective benefits more salient, thus addressing the misconceptions regarding transmission risk that fuel HIV stigma [14]. Furthermore, the authors stress the need for unequivocal messaging which leaves no room for doubt—for example, the presentation of U = U as “100% effective” [6, p.8]. To date, no research has attempted to experimentally compare the differential effects of risk-framed and protection-framed U = U messaging on stigmatising attitudes towards people living with HIV.

Much is known concerning the effects of health message framing on attitudes towards stigmatised groups. For example, Frederick et al. [15] demonstrated that people who read messages which framed obesity in a negative light expressed greater discriminatory attitudes towards people living with obesity, and a higher perceived health risk of living with obesity. Prospect Theory [16] asserts that an individual is inclined to appraise a message in different ways, based on whether the message is framed in terms of the gains (benefits or rewards) or losses (problems or risks) that are associated with the message. Under gain-framed messages, the information is framed in terms of rewards or benefits associated with an outcome, whereas under loss-framed messages, the information is framed in terms of a reduction in an outcome. While the information presented in both cases is factually equivalent, this subtle distinction has differential effects; gain-framed messages are more persuasive in prevention messaging, as they decrease future risk. By contrast, loss-framed messages are better suited in detection messaging, as they are associated with running the risk of detecting illness [17]. Given that the aim of U = U is to decrease risk perception and highlight the benefits of maintaining an undetectable viral load, it is therefore plausible that gain-framed messages that focus on the *protection* afforded by undetectability could lead to more positive perceptions surrounding U = U and PLHIV than loss-framed messages that centre around the *risk* of HIV transmission.

The central objective of this research is to investigate in a nationally representative United Kingdom sample whether a protection-framed message emphasising the protective benefits of an undetectable viral load is more effective at decreasing HIV stigma and increasing perceived accuracy of U = U than a risk-framed message emphasising the reduction in transmission risk afforded by an undetectable viral load.

## Method

### Design

**Fig. 1:**
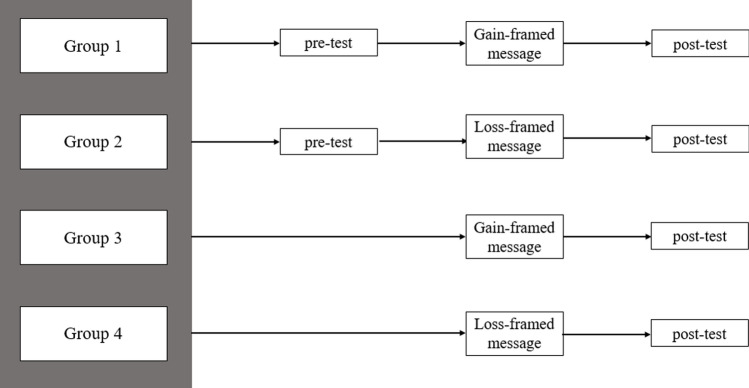
Solomon four-group design

The present study was an online experiment using a variation of the Solomon four-group design [18] to control for pre-test sensitisation effects. In this design, as illustrated in Fig. 1, four groups were formed according to block randomisation, under which participants were randomised to one of the two levels of each independent variable (IV): message group (protection-framed, risk-framed), and test group (pre-test & post-test, post-test only). The Solomon four-group design controls for many other threats to internal validity, such as statistical regression and selection biases, as participants are randomly assigned to one of the four groups [19]. In addition, the design allows for the interaction of pre-test assignment and the independent variable to be studied by employing two groups lacking a pre-test, and as such enhances generalisability [20].

The first dependent variable (DV) was perceived accuracy of U = U, operationalised as response to a 10-point accuracy rating of U = U. The second DV was HIV stigma, operationalised as post-test scores obtained on the HIV/AIDS Behavior Change Communication Toolkit. The covariate was health literacy, operationalised as total score obtained on the 12-item European Health Literacy Survey Short-Form. This covariate was selected due to its documented association with HIV-related knowledge [21]. The pre-registration for this experiment is available on the Open Science Framework: 10.17605/OSF.IO/XFGD7.

### Participants

An a-priori Sample Size Calculator for structural equation models by Daniel Soper [22] was used to conduct a power analysis. A two-tailed alpha of 0.05 was assumed for all tests. Adhering to Cohen’s [23] criteria for small (*w* = 0.1), medium (*w* = 0.3) and large (*w* = 0.5) effect sizes, the smallest effect size of interest, a small-medium effect size of *w* = 0.025 was anticipated, based on a literature review of observed effect size values within previous applications of Prospect Theory to sexual health messaging [24, 25]. Therefore, with a power of 0.8, 2 latent variables, and 15 observed variables, the recommended minimum sample size was 538.

Based on the above estimates, a nationally representative sample of *N* = 700 residents of the United Kingdom was requested from the sample provider, Prolific (prolific.co). To incentivise participation, participants were given £1.10 for having completed the survey; monetary reward size was determined by the sample provider’s algorithm, which considers survey length and sample size. This platform was chosen due to its active userbase (over 67,000 active users as of June 2021). The sample provider used cross-stratified quota sampling to ensure a nationally representative sample of UK residents aged ≥ 18 years, according to gender, age and ethnicity (based on census data).

### Measures

#### Health Literacy

The European Health Literacy Survey Short Form (EHLS) [26] is a 12-item measure of health literacy. Responses are on a 4-point Likert scale, ranging from 1 (very difficult) to 4 (very easy). The scale has a test range of 12–48, in which 48 represents a high level of health literacy. The short-form version of the EHLS has previously demonstrated high internal consistency (Cronbach’s α = 0.87) and satisfactory convergent validity (item-scale correlation ≥ 0.40) [27]. In the present study, the EHLS demonstrated good internal consistency (Cronbach’s α = 0.85). Confirmatory factor analysis was performed to assess construct validity through convergent validity. Convergent validity was assessed using the average variance extracted (AVE) and composite reliability (CR) values. Based on established cut-off values of 0.5 and 0.6 for AVE and CR respectively [28], there was mixed evidence in this study for convergent validity of this measure (AVE = 0.33, CR = 0.85).

#### Stigmatising Attitudes Towards People Living with HIV

An adapted version of a workers’ survey from the *HIV/AIDS Behavior Change Communication Toolkit* [29] was used to measure stigmatising attitudes towards PLHIV. Responses are rated on a 5-point Likert scale, ranging from 1 (strongly agree) to 5 (strongly disagree). There are 44 items in the full scale; the six-item subscale concerning attitudes towards people living with HIV was selected as it is the only subscale to specifically measure stigmatising attitudes, and to reduce participant burden. The language was adjusted to remove stigmatising language (i.e., “infected with HIV” was changed to “living with HIV”). The scale has a test range of 6–30, with 30 representing high stigmatising attitudes. While this measure has not been subject to validation thus far, it was chosen due to a lack of available measures of HIV stigma that have been developed for HIV-negative samples. In the present study, the measure demonstrated excellent internal consistency at both pre-test and post-test (Cronbach’s α = 0.90). Convergent validity was achieved at pre-test (AVE = 0.61, CR = 0.90). and at post-test and (AVE = 0.61, CR = 0.90).

#### Perceived Accuracy of Undetectable = Untransmittable

Participants were presented with the following statement: “*With regard to HIV-positive individuals transmitting HIV through sexual contact, how accurate do you believe the slogan “Undetectable = Untransmittable” is*?” We asked participants to rate the extent to which they believed that the slogan was accurate on a 10-point Likert scale, in which 1 indicated a perception of U = U as highly inaccurate, and 10 indicated a perception of U = U as highly accurate. An alternative option, “*I don’t know what Undetectable = Untransmittable means”* was also provided. Perceived accuracy was assessed at post-test only.

#### Understanding of Undetectable = Untransmittable

After viewing the U = U messages, we asked participants “*If a person living with HIV maintains an Undetectable viral load by regularly taking effective medication, how much protection would this provide against HIV transmission?*”. Responses were on a 5-point Likert scale, ranging from 1 (no protection at all) to 5 (complete protection). An alternative option, “I don’t know what an Undetectable viral load means”, was also provided.

#### Awareness of Undetectable = Untransmittable

After viewing the U = U posters, participants were asked the following question: “*People Living with HIV who maintain an Undetectable viral load by regularly taking effective medication cannot pass on HIV to their sexual partners. Did you know this prior to taking part in this survey?”.* They were asked to indicate their level of prior familiarity with U = U with response options comprising “I knew this already”, “I was unsure of this”, “I didn’t know this already”. Two further options were also available: “I don’t believe this” (to capture those who disagreed with the question posed, regardless of prior awareness), and “I don’t understand this”.

### Procedure

#### Study Procedures

The survey was piloted with a member of the community health worker experienced in HIV support. Data collection took place on 21 June 2021. Prolific users who elected to participate in the study were redirected to a survey hosted by Qualtrics. Upon reaching the survey, participants were presented with a welcome message, and subsequently viewed a Participant Information Sheet. After reading the Participant Information Sheet and providing informed consent, participants completed demographic measures of age, gender, and sexual orientation, and completed the above measure of health literacy. In the first block randomisation, participants were randomly assigned to complete either a pre-test measure of stigmatising attitudes, or not to complete the pre-test. In the second block randomisation, participants were shown one of two U = U messages (available on the OSF project for this study, see “Design”). The messages were presented in poster format, consisting of clear, emboldened text presented against a plain background. The message described U = U as being 100% effective in either “*completely protecting against*” or “*eliminating the risk of*” HIV transmission. To enhance accessibility, the poster was optimised depending on whether the participant was viewing the poster on a PC or a mobile device. Participants were then asked to complete the post-test measures, and subsequently directed to the debriefing message. The procedure was approximately 12 min in duration.

#### Research Ethics and Research Governance

An application for ethical approval was submitted to the School of Psychology Research Ethics Committee at National University of Ireland, Galway on January 8th, 2021 and was approved on January 20th, 2021. If participants had been affected by any issues raised during the survey, they were provided with links to organisations where they could avail of support. Furthermore, while it was possible that the measure of HIV stigma could have invoked feelings of discomfort or mild stress, this possibility was raised in the Participant Information Sheet. Study data was stored on the lead author’s password-protected computer, so that records could be retained for seven years prior to destruction. To credit participants with their incentive, the sample provider used randomly generated participant IDs; however, it was not possible to link participant IDs to study data.

### Statistical Analysis

Scores on the EHLS and the HIV/AIDS Behavior Change Communication Toolkit were coded and compiled using SPSS 27, constituting the health literacy and HIV stigma variables respectively. Structural equation modelling (SEM) was then used to quantify the effects of message frame and test group on the outcomes of HIV stigma and perceived accuracy of U = U. SEM analyses were conducted using AMOS 27 and maximum likelihood estimation. The maximum likelihood estimation approach to analysis of the Solomon four-group design was implemented over both the 2 × 2 factorial ANOVA and regression approaches, as it allowed for modelling of baseline data, thus estimating missing data values regarding those who did not complete the pre-test measurement. [30]. This method avoids the compromise to statistical power observed in conventional treatments of missing data in either a 2 × 2 factorial ANOVA or regression approach, such as in listwise deletion [31]. Model modification indices for structural equation models were not obtained, and model modification not performed, since measurement models were pre-specified on the Open Science Framework. Pre-test data from participants who were not assigned to complete the pre-test (*n* = 354) was treated as missing and estimated using maximum likelihood estimation.

Model 1 included the binary message frame variable, in which participants viewed either a *protection*-framed or a *risk*-framed U = U message, as a predictor of post-test HIV stigma scores, the binary test group variable (pre-test & post-test, post-test only), and the interaction term between the two predictors. Model 2 included perceived accuracy as the primary outcome variable. The effect of the independent variables on HIV stigma and perceived accuracy of U = U, with the mean-centred covariate of health literacy included, were analysed in Model 3 and Model 4, respectively.

## Results

### Descriptive Statistics

A total of 727 individuals viewed the Participant Information Sheet for the study on the host platform. Of these, 713 (98.07%) provided their informed consent, and 707 (97.25%) completed the survey in its entirety—these 707 participants constituted the final analytic sample. Demographic data is presented in Table 1. Transgender male participants were included under ‘males’, and trans women were included under ‘females’.

**Table 1:** Summary of demographic information on final analytic sample (*N* = 707)

	Range	M (SD)
Age	18–80	44.36 (15.38)

	*n*	%
Gender		
Females	362	51.20
Males	342	48.40
Non-binary	2	0.30
Other	1	0.10
Sexual orientation		
Heterosexual	627	88.70
Bisexual	39	5.50
Homosexual	35	5.00
Prefer not to say	3	0.40
Pansexual	2	0.30
Queer	1	0.10
Knowledge of U = U prior to participation		
Did not know of U = U	407	57.60
Was unsure of U = U	172	24.30
Knew of U = U	82	11.60
Does not believe U = U	34	4.80
Does not understand U = U	12	1.70

Means and standard deviations for these variables, stratified by message group and across the total sample, are presented in Table 2, alongside means and standard deviations for these variables stratified by pre-test assignment. Information on model fit for all measurement models, and normality of residuals of all measured variables, can be found as a supplementary material on the OSF Project for this study.

**Table 2:** Means and standard deviations across groups, and parameter estimates for Models 1 & 2 (confirmatory analyses) obtained using maximum likelihood estimation

	Gain-framed message (*n* = 342)		Loss-framed message (*n* = 365)		Total (*N* = 707)	
	*M*	*SD*	*M*	*SD*	*M*	*SD*
Total sample						
Perceived accuracy	5.44	2.96	5.56	2.80	5.50	2.88
HIV stigma post-test	14.37	5.13	14.36	5.26	14.36	5.19
Health literacy	34.89	5.03	34.65	4.83	34.76	4.92
Understanding of U = U	3.71	1.27	3.56	1.26	3.63	1.27
Pre-test-post-test group (*n* = 353)						
HIV stigma pre-test	15.45	5.50	15.13	5.28	15.28	5.38
Perceived accuracy	5.52	3.00	5.91	2.68	5.73	2.83
Health literacy	34.96	4.88	34.76	4.83	34.85	4.85
Understanding of U = U	3.78	1.27	3.68	1.17	3.73	1.21
Post-test only group (*n* = 354)						
Perceived accuracy	5.37	2.92	5.18	2.89	5.28	2.90
Health literacy	34.82	5.17	34.52	4.83	34.67	5.00
Understanding of U = U	3.65	1.27	3.43	1.35	3.54	1.31

	*B*	*β*	*p*
Model 1: HIV stigma			
HIV stigma ← test group	− 0.07	− 0.06	0.102
HIV stigma ← message frame	− 0.03	− 0.03	0.456
HIV stigma ← test group × message frame	0.03	0.02	0.605
Model 2: perceived accuracy of U = U			
Perceived accuracy ← test group	0.70	0.12	0.016
Perceived accuracy ← message frame	0.19	0.03	0.513
Perceived accuracy ← test group × message frame	− 0.52	− 0.08	0.214

*B* unstandardised regression coefficient, *β* standardised regression coefficient

### Confirmatory Analyses

Unstandardised and standardised parameter estimates, standard errors, and *p* values for Model 1 were calculated as part of path analysis in AMOS. According to the results, there was no evidence of an effect of message frame, suggesting no significant difference in post-test HIV stigma scores between participants who viewed a protection-framed U = U message, and those who viewed a risk-framed U = U message [*β* = − 0.03 *p* = .456, 95% CI (− 0.11, 05)]. There was also no evidence of an effect of test group on post-test HIV stigma, suggesting no significant difference between those who completed a pre-test measure of HIV stigma, and those who did not complete the pre-test [*β* = − 0.06, *p* = .102, 95% CI (− 0.14, 0.02)]. There was no evidence of an interaction effect between message frame and test group in this model [*β* = 0.02, *p* = .605, 95% CI (− 0.10, 0.14)]. Pre-test HIV stigma scores had a positive effect on participants’ subsequent post-test scores [*β* = 0.92, *p* < .001, 95% CI (0.81, 1.03)]. Full parameter estimates (unstandardised and standardised regression weights) can be found in Table 2. The supplementary materials, available on the OSF project for this study, provides a visual illustration of each structural equation model and the standardised regression weights for each path.

Unstandardised and standardised parameter estimates, standard errors, and *p* values were calculated for Model 2. According to the results, there was no evidence of an effect of message frame on perceived accuracy of U = U [*β* = 0.03, *p* = .513, 95% CI (− 0.54, 0.61)], suggesting no significant difference in perceived accuracy of U = U between participants who viewed a protection-framed U = U message, and those who viewed a risk-framed U = U message. There was a significant positive effect of test group on perceived accuracy [*β* = 0.12, *p* = .016, 95% CI (0.10, 0.15)], indicating that participants who did not complete the pre-test measure of HIV stigma rated U = U as more accurate on average than those who complete both the pre-test and post-test. There was no evidence of an interaction effect in this model [*β* = − 0.08, *p* = .214, 95% CI (− 0.89, 0.73)].

### Exploratory Analyses

To control for the potentially confounding variable of health literacy, both models were run with the covariate of health literacy added. The results can be viewed within the supplementary materials, available on the OSF project for this study.

To examine whether understanding of U = U moderated the effect of message frame and test group on post-test HIV stigma scores and perceived accuracy of U = U, the primary dependent variables, and the moderator variable, understanding of U = U, were centred, and interaction terms between understanding of U = U and the two primary independent variables were created. The moderator variable was covaried with the primary independent variables and the interaction terms.

According to the results of the maximum likelihood estimation for HIV stigma, there was a positive interaction effect between understanding of U = U and message frame, whereby participants who viewed a protection-framed U = U message reported lower post-test HIV stigma when their understanding of U = U was higher [*β* = 0.08, *p* = .042, 95% CI (0.01, 0.16)]. There was no interaction between test group and understanding of U = U in this model [*β* = − 0.03, *p* = .371, 95% CI (− 0.11, 0.05)]. With respect to the results for perceived accuracy of U = U, there was no evidence for an interaction between understanding of U = U and message frame [*β* = 0.02, *p* = .694, 95% CI (− 0.06, 0.10)] or between understanding of U = U and test group [*β* = − 0.09 *p* = .056, 95% CI (− 0.03, 12)] on perceived accuracy of U = U.

## Discussion

This study demonstrates that in a predominantly heterosexual, nationally representative sample of the UK population, over half of participants had no awareness of U = U prior to their participation, and only approximately one in ten knew about U = U. An overall favourable response to the U = U messages presented was found in this study, with the majority of participants rating U = U as at least somewhat accurate, as quantified by a median accuracy rating of 6/10. Not only is this in line with other contemporary data on the perceived accuracy of U = U [5, 7], this finding is promising given that the above studies have largely sampled gay, bisexual and other men who have sex with men (gbMSM), who are considered as more engaged with, and more receptive to, U = U messaging.

The primary aim of the present study was to empirically test whether a protection-framed Undetectable = Untransmittable (U = U) message is more effective at decreasing HIV stigma and increasing U = U perceived accuracy than a risk-framed message. A message emphasising the protective benefits of U = U (*protection-*framed message) was contrasted with a message which emphasised U = U’s ability to completely reduce HIV transmission (*risk*-framed message), among a large, nationally representative sample in the United Kingdom.

There were no observed differences in either post-test HIV stigma scores or perceived accuracy of U = U between those who viewed a message emphasising the protective benefits of U = U, and those who viewed a message emphasising a reduction in risk. This finding does not seem to support the recommendation by Rendina et al. [5] that U = U messages should use protection-focused language, nor does it align with Prospect Theory literature which posits that gain-focused language is more efficacious when used in messages which communicate illness prevention methods [32]. Plausible reasons for this finding include the intervention provided in this study being light-touch, and the distinction between the messages being subtle. Future studies examining variations in U = U message framing will need to balance highlighting the contrast between message frames with ensuring that messages frames are not disparate enough to introduce confounding influences on persuasiveness.

Exploratory analyses also yielded several interesting insights; when controlling for health literacy, there were no significant differences between either HIV stigma or perceived accuracy of U = U (see “Design” for supplementary materials). This suggests that an individual’s ability to understand health-related information did not confound the relative persuasiveness of the protection-framed message or the risk-framed message. However, irrespective of framing, higher health literacy scores were associated with more favourable ratings of U = U. This highlights the need for tailored U = U messaging which considers differing capacities for health literacy (such as one’s ability to elaborate on a message in a given context). These findings also demonstrate the need to communicate U = U in clear terms, as the phrase “Undetectable = Untransmittable”, may not have an intuitive meaning to the casual observer. Further to this, there has been documented misunderstanding of the word “Undetectable” [33]. Moderation analysis revealed that participants with a greater understanding of U = U reported lower HIV stigma in the protection-framed condition—however, a lack of acceptable model fit limits the interpretability of this finding.

There was a positive effect of test group on perceived accuracy of U = U in this study, whereby participants who did not complete a pre-test measure of HIV stigma rated U = U as more accurate than those who completed a pre-test measure. This suggests that individuals rated U = U as less accurate having previously been asked to complete a pre-test measure of stigma. Such question-behavior effects (QBEs) constitute a change in a relevant outcome due to the mere questioning of an individual [34], by influencing the accessibility of an attitude [35]. In the present study, the pre-test questionnaire content focused on affective attitudes (feelings or emotions about PLHIV). Questionnaire content emphasising affective attitudes has been identified as a possible source of QBEs [36], whereby emotional beliefs or reactions are more immediately accessible after having viewed such content. Thus, in the present study, if participants held negative implicit attitudes about PLHIV, the questionnaire content would have increased the availability of such attitudes, resulting in lower perceived accuracy of U = U.

One significant advantage of the Solomon four-group design used in this study is it allows for the control of QBEs, and thus bias which could have been introduced by pre-test measurement. This research design could therefore serve as a fruitful method for subsequent HIV stigma experimental research seeking to measure pre- and post-intervention attitudes. Other strengths of the present study are its preregistration, the recruitment of a large, nationally representative sample, and an approach to statistical analysis of the Solomon four-group design which maximised statistical power—thus, many threats to external and internal validity have been protected against.

The main limitation of the present study concerns possible issues surrounding social desirability arising from measurement of constructs such as HIV stigma using self-report measures. Despite the online survey maintaining confidentiality and anonymity, participants may have felt the need to report more favourable attitudes towards PLHIV, knowing they were participating in stigma research. Thus, self-report measures of stigma do not necessarily represent how individuals behave towards PLHIV in the real world, across all types of behaviors. Another limitation concerning measurement relates to the measures of prior awareness of U = U and understanding of U = U in this study. For those who selected “I don’t understand this” for the measure of prior awareness, this group may have been indicating either that they did not understand the question asked, or that they did not understand U = U itself. The latter outcome would introduce overlap between this measure and the measure of understanding (which offered an option to say “I don’t know what an Undetectable viral load means”). Future research examining similar constructs should take care to avoid possible measurement issues by ensuring the data obtained provides easily distinguishable information regarding the attitudes of the respondent.

## Conclusions

With the “Undetectable = Untransmittable (U = U)” campaign having marked its fifth anniversary in July 2021, the findings of the present study are promising, while also providing a clear indication of how U = U is being perceived, understood, and internalised among a large nationally representative UK sample. Findings suggest no evidence of a significant difference between a protection-framed message, which emphasised the protective benefits of U = U, and a risk-framed message, which emphasised a reduction in transmission risk, on HIV stigma or perceived accuracy of U = U. Exploratory analyses revealed health literacy to be positively associated with perceived accuracy of U = U. This study also finds that among a predominantly heterosexual, nationally representative sample in the United Kingdom, the majority rate U = U as at least somewhat accurate following a brief intervention.

## Data Availability

Study materials are available on the Open Science Framework: 10.17605/OSF.IO/XFGD7.
